# mirSNPs as Potential Colorectal Cancer Biomarkers: A Systematic Review

**DOI:** 10.3390/ijms252312975

**Published:** 2024-12-03

**Authors:** Katiusse Alves dos Santos, Lourdes Maria Costa Alves de Sousa, Karla Simone Costa de Souza, Olalla Maroñas Amigo, André Ducati Luchessi, Vivian Nogueira Silbiger

**Affiliations:** 1 Postgraduate Program in Pharmaceutical Sciences, Federal University of Rio Grande do Norte, Natal 59012-570, Brazil; katiusse.santos.059@ufrn.edu.br; 2 Faculty of Pharmacy, Federal University of Rio Grande do Norte, Natal 59012-570, Brazil; maria.costa.708@ufrn.edu.br; 3 Department of Clinical and Toxicological, Federal University of Rio Grande do Norte, Natal 59012-570, Brazil; karlacostasouza@gmail.com (K.S.C.d.S.); andre.luchessi@outlook.com (A.D.L.); 4 Pharmacogenomics and Drug Discovery (GenDeM), Foundation of Health Research Institute of Santiago de Compostela (FIDIS), 15782 Galicia, Spain; olalla.maronas@usc.es; 5 Genomic Medicine Group, Galician Public Foundation for Genomic Medicine (FPGMX), 15782 Galicia, Spain

**Keywords:** mirSNP, miRNA, biomarkers, colorectal cancer

## Abstract

Colorectal cancer (CRC) is the third most common neoplasm in the world and the second with the highest mortality rate. Single nucleotide polymorphisms (SNPs) in microRNA (miRNA) genes known as mirSNPs may be related to dysregulated miRNA expression in several neoplasms. This systematic review aims to investigate studies that investigate SNPs located in regions of miRNA genes that influence their expression and are associated with CRC, as well as their potential as biomarkers for the disease, based on the available literature. For this, searches were performed in public databases, including MEDLINE/PubMed, Embase, Web of Science, and Scopus. The rigorous review of the PRISMA 2020 guidelines and the methodological quality of these studies was assessed using the Newcastle–Ottawa scale and the Mixed Methods Assessment Tool. Of the 175 studies identified, 26 were considered eligible: 18 of them highlighted mirSNPs as potential biomarkers of risk and prognosis for CRC; 4 studies suggested a protective role; 1 study linked mirSNPs to treatment; 3 studies found no relevant evidence. These results highlight the importance of conducting further research on the topic, given the potential of these biomarkers to contribute to risk assessment, prognosis, and the development of therapeutic strategies for patients with CRC.

## 1. Introduction

Colorectal cancer (CRC) is a malignant neoplasm that affects the large intestine and rectum and is notable for ranking third in incidence and the second leading cause of cancer-related deaths worldwide [1]. Most cases of CRC arise because of a combination of genetic and environmental factors, with several elements contributing to an increased risk of development. Modifiable risk factors include diet, obesity, physical inactivity, smoking, and alcohol consumption, while non-modifiable factors include gender, ethnicity, personal history of inflammatory bowel disease, personal or family history of polyps or cancer, hereditary conditions such as Lynch syndrome, and genes involved in carcinogenesis [2]. Current methods of diagnosis and prognostic monitoring of CRC include colonoscopy and flexible sigmoidoscopy; however, these techniques are invasive, uncomfortable, and often suffer from poor patient compliance [3]. Alternative methods such as fecal occult blood tests, fecal immunochemical tests, carcinoembryonic antigen (CEA), and carbohydrate antigen 19–9 (CA19–9) are affected by high false-positive rates and low sensitivity. Each method has distinct advantages and limitations that impact clinical decision-making [4]. In addition to conventional treatments, promising therapeutic approaches are being investigated [5,6,7,8]. There is an increasing focus on research into individualized genetic risk profiles to improve the diagnosis and prognosis of CRC [9,10]. Numerous studies aim to identify molecular markers that may contribute to the development of personalized medicine, improving disease prediction and patient outcomes [11]. Advances in molecular biology techniques increasingly aim to develop methods to obtain accurate information about tumors [12].

Among the widely studied markers, microRNAs (miRNAs) stand out for their differential expression in tumors and their presence in body fluids, together with their stability under different storage and handling conditions [13,14,15,16]. MiRNAs are small non-coding RNAs, typically approximately 22 nucleotides in length, that regulate gene expression by binding to complementary sites within the 3′-untranslated regions (3′-UTRs) of target mRNAs, thereby inhibiting mRNA translation [17]. These molecules play a crucial role in cancer biology, influencing processes such as cell proliferation, differentiation, migration, angiogenesis, and apoptosis. Dysregulated expression of miRNAs has been associated with the development and progression of CRC, with miRNAs acting as oncogenes or tumor suppressors [18]. MiRNAs play a crucial role in monitoring CRC, as their expression varies significantly at different stages of the disease. This characteristic allows tracking its progression and assessing response to treatment. Furthermore, early detection of CRC remains essential to reduce mortality, given that survival rates are strongly linked to the stage at which cancer is diagnosed. This makes them potential markers of diagnostic accuracy and effective tools for monitoring disease progression and response to treatment [17,18].

Furthermore, single nucleotide polymorphisms (SNPs) in miRNA-associated regions such as the 3′-UTR, known as mirSNPs, can alter the binding affinity between miRNAs and target mRNAs, leading to changes in miRNA expression and maturation, as well as changes in target gene expression levels [19,20]. SNPs can be found in different miRNA-related regions, such as pre-miRNA, mature miRNA, seed region, or miRNA binding sites, significantly impacting miRNA function and contributing to susceptibility to several diseases, including cancer [21,22]. In this systematic review, we emphasize SNPs located in miRNA binding sites associated with CRC, exploring their potential as biomarkers for this disease in alignment with the existing literature.

## 2. Methods

### 2.1. Protocol and Registration

This systematic review follows the Preferred Reporting Items for Systematic Reviews and Meta-Analyses (PRISMA) guidelines (Table S1) [23]. The review protocol was registered in the International Prospective Register of Systematic Reviews (PROSPERO) under number CRD42024574543. Following registration, changes to the protocol were made and recorded and can be accessed at https://www.crd.york.ac.uk/prospero/display_record.php?ID=CRD42024574543 (accessed on 31 October 2024).

### 2.2. Inclusion and Exclusion Criteria

Eligibility criteria were carefully defined to identify studies addressing SNPs located in miRNA binding sites in target genes associated with CRC. This review included observational and clinical studies published without date restrictions, peer-reviewed, in English, and available in scientific journals. Review studies, systematic reviews, books, case reports, short communications, editorials, letters to the editor, preprints, conference proceedings, dissertations, theses, preclinical studies, and studies addressing SNPs in lcnRNAs were excluded.

### 2.3. Literature Search Strategy

We conducted a systematic search for original articles in the literature in July 2024, using the MEDLINE/PubMed, Embase, Scopus, and Web of Science databases. The search strategy employed a wide range of keywords, aligned with the study objectives, and Boolean operators (AND, OR, NOT) were strategically employed to refine the search terms. The following terms were used: “colorectal cancer mirSNP” OR “neoplasm colorectal mirSNP” AND “miRNA SNP colorectal cancer” OR “miRNA SNP colorectal neoplasm.” After the search, the articles were imported into the RAYYAN software version 2024, accessed at https://www.rayyan.ai (accessed on 24 August 2024), for duplicate removal. Two authors (KAS and LMCAS) independently screened the studies by evaluating titles and abstracts based on predefined eligibility criteria. After the initial screening, the full texts of the articles were reviewed by both researchers independently for final inclusion. In case of discrepancy between researchers, the opinion of a third author (KSCS) was requested.

### 2.4. Data Collection

**Figure 1:**
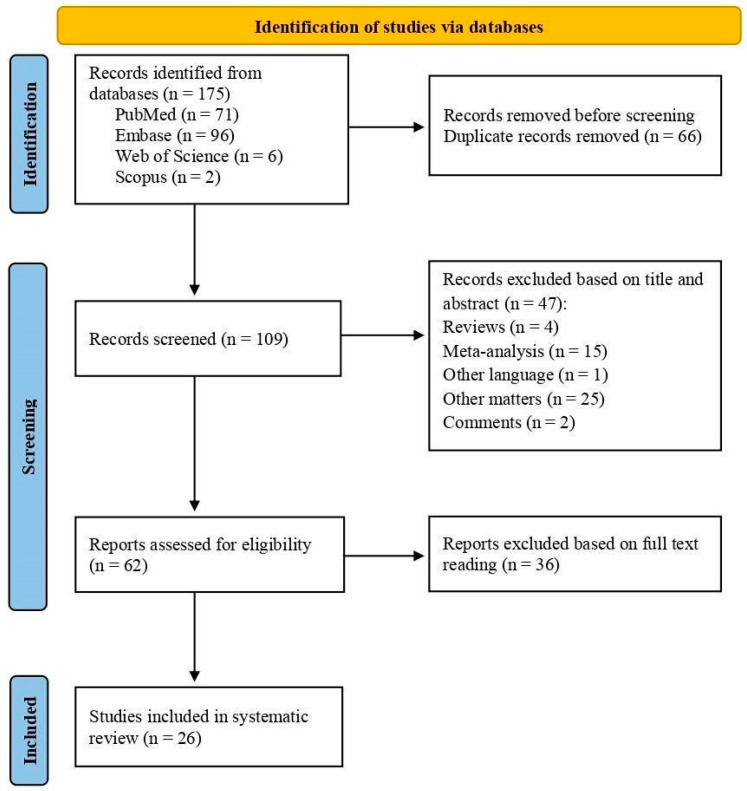
The flowchart illustrates the article selection methodology according to the recommendations of the Systematic Reviews and Meta-Analyses (PRISMA) [23].

The bibliographic search in the databases resulted in 175 records. After eliminating 66 duplicates, 109 titles, and abstracts were evaluated by two independent reviewers. Of these, 47 studies were excluded for not meeting the inclusion and exclusion criteria, leaving 62 studies for full reading. Among these, 36 studies were discarded for not meeting the eligibility criteria. As a result, 26 articles were considered eligible and included in the systematic review, as illustrated in the article screening process flowchart (Figure 1). Information about authors, year of publication, study type, study population, miRNA SNP location, altered miRNA, SNP rsID, target gene, and miRNA SNP effect on CRC were extracted from each study when available (Table 1).

### 2.5. Quality Assessment and Risk of Bias

Two reviewers (KAS and LMCAS) independently assessed the methodological quality and risk of bias of the studies included in the systematic review. The Mixed Methods Assessment Tool (MMAT) [24] was used for this assessment, as detailed in Table S2 [25,26,27,28,29,30,31,32,33,34,35,36,37,38,39,40,41,42,43,44,45,46,47,48,49,50]. The adapted Newcastle–Ottawa scale [51] was used to assess the risk of bias in case-control and cohort studies, with scores classified as very high risk of bias (0–3), high risk (4–6), or high methodological quality (7–9) (Table S3). Discrepancies in assessment were resolved through discussion between the reviewers or, if necessary, by consulting a third researcher.

**Table 1:** General characterization of included studies.

Study Design	Author	Population	SNP ID	Allele/ Genotype	Gene ID	miR ID	Association of mirSNPs with CRC
Case-control study	[25]	Czech Republic	rs17281995 rs1051690	G>A	CD86 INSR	miR-337, miR-582, miR-200a, miR-184, and miR-212 miR-618 and miR-612	Increased risk
Case-control study	[26]	Chinese	rs141178472	CC	PIK3CA	miR-520a	Increased risk
Case-control study	[27]	Chinese	rs3814058	C>T	PXR	hsa-miR-129-5p	Increased risk
Case-control study	[28]	Chinese	rs6504593	T	IGF2BP1	miR-21	Increased risk; cell proliferation and apoptosis
Case-control study	[29]	Chinese	rs1062044	A	LAMC1	miR-423-5p	Increased risk; colon adenocarcinomas
Case-control study	[30]	Korea	rs7930	AG	TOMM20	miR-4273-5p	rs7930 A>G: Increased risk; Suppression of TOMM20 gene expression
Case-control study	[31]	Spain	rs868		TGFBR1	let-7	Increased risk
Case-control study	[32]	United States	rs8176318 rs8905	AA GG	BRCA1 PRKAR1A	miR-525-5p miR-miR-214-3p	Increased risk
Case-control study	[33]	Tunisian	rs2292832 rs2910164	C/T G/C		hsa-mir-149 hsa-mir-146a	Increased risk
Case-control study	[34]	Chinese	rs895819	GG		miR-27a	Increased risk
Case-control study	[35]	Chinese	rs2910164	G>C		miR-146a	Susceptibility and differentiation
Cohort Studies	[36]	Korea	rs3757417 rs12373	T>G A>C	TPST1 PAUF	miR-571	Prognostic markers
Case-control study	[37]	Iran	rs12904	AA/A>G	EFNA1	miR-200C and miR-429	rs12904 AA: Pathogenesis and metástasis; rs12904 A>G: reduced disease progression; regulation of EFNA1 gene expression.
Cohort Studies	[38]	Dutch	KRAS-LCS6	T>G	KRAS	let-7	Prognostic biomarker
Case-control study	[39]	Caucasian and African-American	rs4919510	GG		hsa-miR-608	Increased risk of death in Caucasians and a reduced risk in African Americans
Case-control study	[40]	Chinese	rs11614913	CC		miR-196a2	Increased susceptibility in patients who have undergone chemotherapy treatment
Cohort Studies	[41]	Chinese	rs2273626 rs202195689	A>C CCCCA>del		hsa-miR-4707-3p hsa-miR-4274	Predictive and prognostic biomarkers after chemoradiotherapy
Case-control study	[42]	Chinese	rs2682818	AA and AC/AA		miR-618	Reduction in risk
Case-control study	[43]	Chinese	rs187960998	C>T	CHD5	miR-211	Decreased risk and protective factor for CC by preventing binding of the tumor suppressor gene CHD5
Case-control study	[44]	Bulgaria	rs2682818	AC		miR-618	Decreased risk of susceptibility to mCC
Case-control study	[45]	Chinese	rs35301225	C/A	E2F1	miR-34a	Tumor suppression and protection by downregulating the E2F1 gene; rs35301225 C>A: upregulation of E2F1 expression, resulting in worse survival.
Case-control study	[46]	Korea	rs2279398	G>A	DOK3	miR-370	Reduction in risk
Randomized study	[47]	Multicenter study	rs4919510	CC		miR-608	Prognostic biomarker
Case-control study	[48]	Chinese	rs11614913	T>C		miR-196a2	No significant association with increased risk or disease progression was identified
Case-control study	[49]	Iran	rs11614913			miR-196a2	No significant association was demonstrated between the SNP rs11614913 in miR-196a2 and CRC
Case-control study	[50]	Iran	rs12904		EFNA1	miR-200c	No significant association was demonstrated between rs12904 in miR-200c and CRC risk

## 3. Results

This review analyzed 26 studies conducted between 2008 and 2022 on SNPs that affect miRNA expression and their influence on the occurrence and progression of CRC in different populations. Most studies adopted a non-randomized quantitative design (96.15%), with emphasis on research conducted predominantly in the Chinese population [26,27,28,29,34,35,40,41,42,43,45,50]. The most frequently cited miRNA was miR-618 [25,42,44]. Of note, the SNP rs1051690 located in the miR-618 target gene region was associated with an increased risk of CRC in one study. In contrast, the SNP rs2682818 in mature miR-618 was correlated with a reduced risk. These discrepancies highlight that, in the case of miR-618, different SNPs (rs1051690 and rs2682818) may affect its expression and activity in distinct ways, resulting in opposite effects on CRC risk, as evidenced by the different results of the studies analyzed.

Seventeen studies highlighted mirSNPs as potential biomarkers of CRC risk and prognosis. Among the mirSNPs associated with CRC risk, the following were identified: SNP rs17281995 (miR-337, miR-582, miR-200a, miR-184, and miR-212) and rs1051690 (miR-618 and miR-612) [25], rs141178472 (miR-520a) [26], rs3814058 (hsa-miR-129-5p) [27], rs6504593 (miR-21) [28], rs1062044 (miR-423-5p) [29], rs7930 (miR-4273-5p) [30], rs868 (let-7) [31], rs8176318 (hsa-miR-525-5p), rs8905 (hsa-miR-214-3p) [32], rs2292832 (hsa-mir-149), rs2910164 (hsa-mir-146a) [33], and rs895819 (miR-27a) [34].

The highlighted mirSNPs associated with prognosis were: rs2910164 (miR-146a) [35], rs12373 (miR-571) [36], rs12904 (miR-200C and miR-429) [37], KRAS-LCS6 (let-7) [38], rs4919510 (hsa-miR-608) [39], rs11614913 (miR-196a2) [40], rs2273626 (hsa-miR-4707-3p), and rs202195689 (hsa-miR-4274) [41]. Furthermore, five studies identified mirSNPs as potential protective biomarkers: rs2682818 (miR-618) [42], rs187960998 (miR-211) [43], rs2682818 (miR-618) [44], rs35301225 (miR-34a) [45] and rs2279398 (miR-370) [46]. One study reported a mirSNP associated with the treatment of CRC patients: rs4919510 (miR-608) [47]. Finally, three studies found nonsignificant associations between mirSNPs and CRC: rs11614913 (miR-196a2) [48,49] and rs12904 (miR-200c) [50] (Table 1).

### 3.1. SNPs in Target Genes and Mature miRNAs Associated with Colorectal Cancer Risk

Ten studies highlighted CRC risk-associated SNPs located in miRNA target gene regions [25,26,27,28,29,30,31,32,33], while one study investigated SNPs located in mature miRNA regions [34] (Table 2). The earliest study, conducted by Landi et al., included 697 CRC cases and 624 controls in the Czech Republic. This study identified two relevant SNPs: rs17281995 in the Cluster of Differentiation 86 (CD86) gene and rs1051690 in the insulin receptor (INSR). Both SNPs affect miRNA binding sites and are associated with an increased CRC risk. Furthermore, the rs1051690 G>A genotype in the 3′-UTR of INSR demonstrated a significant biological impact on the target miRNA [25]. In a study conducted by Ding et al. with 386 cases and 394 controls in China, it was identified that the SNP rs141178472, located in the miR-520a binding site of the phosphatidylinositol-4,5-bisphosphate 3-kinase catalytic subunit alpha gene (PIK3CA), is associated with the risk of CRC. Individuals with the rs141178472 CC genotype had a significantly higher risk of CRC compared with those with the rs141178472 TT genotype [26]. In the same year, Ni et al. performed an in vitro experiment involving 1033 CRC cases and 1147 controls from the same Chinese population. The results suggested that the SNP rs3814058 C>T in the target site of hsa-miR-129-5p may influence the expression of the pregnane X receptor (PXR) gene, increasing the susceptibility to the development of CRC [27]. Another study conducted in the Chinese population by Xie et al. analyzed 1147 individuals with CRC and 1203 controls in a case-control study. The authors found that the SNP rs6504593, located in the miR-21 binding site in insulin-like growth factor 2 (IGF2BP1) mRNA-binding protein, may modify the expression of this gene and contribute to the risk of CRC. In vitro analysis revealed that the T allele of rs6504593 negatively regulates the post-transcriptional expression of IGF2BP1 by altering the binding affinity of miR-21. The inhibition of miR-21 led to decreased cell growth, while miR-21 mimetics promoted cell proliferation and suppressed apoptosis [28]. In a study conducted by Ke et al. with 2347 cases and 3390 Chinese controls, it was demonstrated that the SNP rs1062044 influences the interaction between miR-423-5p and Laminin Subunit Gamma 1 (LAMC1). The carriers of the rs1062044 A allele had a significantly higher risk of CRC. Additionally, a negative correlation between the expression levels of miR-423-5p and LAMC1 was observed in patients with colon adenocarcinomas [29]. A study conducted in Korea involving 325 CRC patients and 977 controls identified the rs7930 SNP in miR-4273-5p as a potential risk factor for the disease. According to the results of Lee et al., individuals carrying the rs7930 AG genotype had an increased risk of CRC compared with those with the rs7930 AA genotype. This increased vulnerability is associated with the specific interaction of miR-4273-5p with the A allele of the rs7930 SNP, which leads to the suppression of the expression of the TOM20 mitochondrial import receptor subunit gene (TOMM20). Thus, the A allele is considered protective, while carriers of the G allele have an increased risk of developing CRC [30]. Additionally, a study conducted in Spain investigated the association between the SNP rs868, located in the miRNA let-7 binding site in the 3′-UTR of the transforming growth factor beta receptor 1 gene (TGFBR1). The results demonstrated that the presence of this SNP increases the binding of TGFBR1 to the miRNA let-7b-5p, resulting in decreased TGFBR1 expression and, consequently, increasing the risk of CRC [31]. Mullany et al. also explored the influence of altered miRNA expression in SNPs located in target gene regions and their impact on cancer risk. The study revealed that two specific SNPs rs8176318 AA, located in the 3′-UTR of the Breast Cancer 1 (BRCA1) gene, predicted to bind to miR-525-5p, and rs8905 GG, which targets the cAMP-dependent protein kinase type I regulatory subunit alpha (PRKAR1A) in miR-214-3p, were significantly associated with the risk of colon cancer (CC) in the United States population [32]. Chayeb et al. sought to clarify whether the SNPs rs2292832 C>T in hsa-mir-149, rs2910164 G>C in hsa-mir-146a, and rs11614913 C>T in hsa-mir-196a2 are associated with the risk and prognosis of CRC. The study included 152 CRC patients and 161 Tunisian controls, and the results revealed a significant association between the SNPs rs2292832 C>T in hsa-mir-149 and rs2910164 G>C in hsa-mir-146a with the risk of CRC, considering ethnicity; however, no significant association was found for rs11614913 C>T in hsa-mir-196a2 [33]. In a complementary study carried out by Cao et al. with 254 CRC patients and 238 healthy controls from the Han Chinese population, the relationship between the expression of the SNP rs895819 in miR-27a and the risk of CRC was examined. The results showed that patients with rs895819 AG/GG genotypes had a significantly higher risk of CRC compared with those with the rs895819 AA genotype. Furthermore, further analysis indicated that miR-27a was more highly expressed in the tumor tissue samples of patients with the GG genotype relative to those with the rs895819 AA genotype [34].

### 3.2. SNPs in miRNA Target Genes, Complementary Regions, Mature miRNA, and Seed Regions Associated with Colorectal Cancer Prognosis

Three studies have identified SNPs associated with CRC prognosis in miRNA target gene regions [35,36,37]. One study focused on complementary regions [38], while two analyzed mature miRNA regions [39,40], and another studied a SNP located in the seed region [41] (Table 3). In a study conducted in China, Hu et al. investigated the effects of SNPs rs2910164 and rs3746444, located in miR-146a and miR-499, respectively, in 276 cases and 373 healthy Chinese individuals. The results demonstrated that the SNP rs2910164 G>C in miR-146a is associated with CRC susceptibility and differentiation, while the SNP rs3746444 in miR-499 showed no significant association with the disease. The authors highlighted the SNP rs2910164 in miR-146a as a potential biomarker for CRC initiation and progression [35]. In the same year, Kim et al. conducted a study with 831 Korean individuals diagnosed with CRC. The analyses demonstrated that the SNPs rs3757417 T>G and rs12373 A>C in the 3′-UTR of Tyrosylprotein Sulfotransferase 1 (TPST1) and pancreatic adenocarcinoma upregulated factor (PAUF), respectively, are considered potential prognostic markers for patients undergoing surgical resection. The authors also reported that the SNP rs12373 A>C increases the binding affinity of miR-571, resulting in the suppression of PAUF gene expression, which may impact the survival of CRC patients [36]. In a case-control study conducted by Salem et al. involving 146 CRC patients and 132 healthy individuals from Iran, it was identified that the rs12904 AA genotype plays a crucial role in the pathogenesis and metastasis of CRC. In contrast, patients carrying the rs12904 A>G genotype showed significantly reduced disease progression. The authors also observed that the SNP rs12904 in the ephrin A1 ligand gene (EFNA1) contains binding sites for the miRNAs miR-200C and miR-429, which may be important in regulating the expression of this gene [37]. Another study, conducted by Smits et al. with a significant number of Dutch individuals, investigated an SNP located in the complementary site of let-7 in the 3′-UTR region of the KRAS gene (KRAS-LCS6). The analyses revealed that the SNP LCS6 T>G in this region is associated with improved survival in patients with early-stage CRC; therefore, the G allele in KRAS-LCS6 can be considered a promising prognostic biomarker for early-stage CRC [38]. The study by Ryan et al. revealed that SNP rs4919510 in the mature sequence of hsa-miR-608 may have a significant impact on the prognosis of CRC patients, analyzing 245 cases and 446 controls from Caucasian and African-American populations. Although the results did not show a significant association between the SNP rs4919510 and CRC risk, a correlation with survival was identified. The rs4919510 GG genotype was associated with an increased risk of mortality among Caucasians, while African-Americans had a reduced risk of death, suggesting that the effect of this SNP may vary by ethnicity [39]. Another study conducted by Zhan et al., which involved 252 CRC patients and 543 healthy individuals from the Chinese population, sought to determine whether the SNP rs11614913 in miR-196a2 is related to CRC progression and susceptibility. The results showed that the rs11614913 CC genotype was more prevalent among CRC patients compared with controls. Furthermore, carriers of the rs11614913 CC genotype, or those with at least one C allele, presented elevated levels of mature miR-196a, which was associated with increased susceptibility to CRC; however, no association with disease progression was observed. The authors also highlighted that such associations were exclusively observed in patients undergoing chemotherapy treatment [40]. Huang et al. observed that the SNP rs2273626 A>C in hsa-miR-4707-3p was significantly associated with a decreased risk of grade 2 leukopenia and the SNP rs202195689 hsa-miR-4274 with overall survival and free survival of disease in patients who received postoperative chemoradiotherapy. These results suggest these mirSNPs as potential predictive and prognostic biomarkers in chemoradiotherapy and clinical care of patients with rectal cancer [41].

### 3.3. SNPs in Target Genes and Mature miRNAs Associated with Reduced Colorectal Cancer Risk and Protection

Three studies highlighted SNPs associated with reduced risk and protection against CRC, located in mature miRNA regions [42,43,44], and two studies focused on target gene regions [45,46] (Table 4). In 2018, Chen et al. analyzed 878 CRC patients and 884 controls from the Chinese Han population of China, investigating the relationship of the SNP rs2682818 in miR-618 with CRC. The results showed that the AA and AC/AA genotypes of rs2682818 were associated with a reduced risk of CRC compared with the rs2682818 CC genotype [42]. In the following year, Zhu et al. conducted a study involving 685 patients with CC and 618 healthy controls belonging to the same population, evaluating the SNP rs187960998, located in miR-211, and its possible relationship with the occurrence of CC. The authors observed a significant association between the rs187960998 CT genotypes and favorable clinical characteristics, such as smaller tumor size, better differentiation, and lower probability of metastasis. Furthermore, the carriers of the rs187960998 CT/TT genotypes and the rs187960998 T allele had a reduced risk of developing the disease. These findings suggest that the SNP rs187960998 in miR-211 may play a protective role by inhibiting binding to the 3′-UTR of the tumor suppressor gene chromodomain helicase DNA-binding protein 5 (CHD5) [43]. In Bulgaria, Radanova et al. investigated the association of the SNP rs2682818 in miR-618 with susceptibility to metastatic colon cancer (mCC) in 104 patients before the initiation of chemotherapy. The results showed that the risk of developing mCC was significantly lower in patients with the heterozygous rs2682818 AC genotype compared with those who had the homozygous rs2682818 CC and rs2682818 AA genotypes [44]. On the other hand, Jiang et al. conducted a study with 685 newly diagnosed CRC patients and 618 controls from the Chinese population, aiming to investigate the association of the SNP rs35301225 in miR-34a with the risk of CRC. The results indicated that the A genotype of the rs35301225 SNP in miR-34a may act as a tumor suppressor by downregulating the oncogenic gene E2F Transcription Factor 1 (E2F1). Thus, the presence of this SNP can be considered a protective factor against CRC; however, the authors also observed that the rs35301225 CA genotype in miR-34a is associated with increased E2F1 expression, which in turn is associated with worse survival in CRC patients [45]. The study by Kang et al. conducted on 895 cases and 901 controls from the Korean population suggested that the SNP rs2279398 G>A, located in the miRNA binding site, may influence the expression of the docking protein 3 (DOK3) gene. This influence is due to the direct binding of miR-370 to the 3′-UTR region of the gene. These findings indicate that this SNP in the DOK3 gene plays a significant role in decreasing the risk of CRC [46].

### 3.4. SNPs in Regions of Mature miRNAs Associated with Colorectal Cancer Treatment

Only one study has identified SNPs associated with CRC treatment located in mature miRNA regions. Sclafani et al. investigated the impact of the SNP rs4919510 on mature miR-608 in a retrospective study involving 155 patients with locally advanced rectal cancer (LARC). The study evaluated the effect of perioperative treatment with Capecitabine and Oxaliplatin (CAPOX) as neoadjuvant therapy, followed by chemoradiotherapy, surgery, and adjuvant treatment with CAPOX and Cetuximab. The results revealed that individuals with the rs4919510 CC genotype had a worse prognosis compared with those with the rs4919510 CG/GG genotypes, especially among patients undergoing systemic chemotherapy, chemoradiotherapy, surgery, and adjuvant chemotherapy [47] (Table 4).

### 3.5. SNPs in Target Genes and Mature miRNAs Not Associated with Colorectal Cancer

Several studies have analyzed the association of mirSNPs with CRC and found no statistical significance in their results. Among them, two focused on SNPs located in miRNA target gene regions [48,49], while one study focused on regions of mature miRNAs [50] (Table 5). Chen et al. investigated the relationship between the SNP rs11614913 in miR-196a2 with susceptibility and progression of CRC. The research involved 126 CRC patients and 407 healthy individuals from the Chinese population, but no significant association with increased risk or disease progression was identified [48]. In 2020, Ayadilord et al. conducted a case-control study with 52 CRC patients and 120 healthy Iranian individuals to evaluate the correlation between SNP rs11614913 in miR-196a2 and CRC. The genotyping results showed no statistically significant association between this SNP and CRC [49]. Another study conducted in Iran involving 152 CRC patients and 160 controls investigated the influence of the rs12904 SNP located in the 3′-UTR region of the EFNA1 gene on the regulation of miR-200c expression and CRC risk. The results also did not demonstrate a significant association between the SNP rs12904 and CRC risk in the analyzed groups [50].

### 3.6. Quality and Risk of Bias of the Evaluated Studies

The methodological quality of the studies included in the systematic review was carefully assessed, and the results can be found in the Supplementary Materials (Tables S2 and S3). Of the 22 case-control studies and 3 cohort studies analyzed, 17 case-control studies and 1 cohort study had scores between 7 and 9, reflecting high methodological quality. In contrast, 5 case-control studies and 2 cohort studies had scores of 4 to 6, raising concerns regarding the risk of bias and the validity of the results.

## 4. Discussion

In this systematic review, we performed a detailed analysis of SNPs in miRNA binding sites associated with CRC, aiming to identify promising markers for early detection, risk prediction, and prognosis of the disease. To achieve this objective, we reviewed 26 studies that met the established inclusion criteria.

SNPs in miRNA regions represent a promising class of genetic variations with great potential to be explored as markers of individual susceptibility to complex diseases, as well as a tool for prognosis and clinical decision support [52]. These variations can impact miRNA-mediated post-transcriptional regulation, leading to changes in the expression of crucial genes. Consequently, these changes may contribute to interindividual variability in the risk of developing cancer [53,54,55]. Furthermore, SNPs located in miRNA target regions in genes relevant to CRC may play a significant role in its etiopathogenesis [56]. SNPs in the pri-miRNA and pre-miRNA regions can affect miRNA processing and maturation, impacting primary transcription and miRNA-target interactions, which may lead to altered expression of mature miRNA [42,57]. Similarly, SNPs in the mature sequence or seed region of the miRNA can modify interactions with target sites, altering the binding affinity between miRNAs and their target mRNA [39].

**Figure 2:**
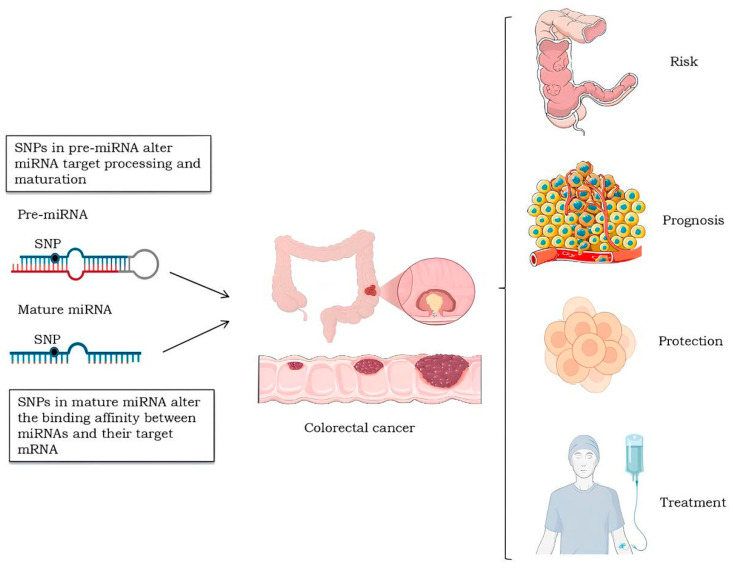
Single nucleotide polymorphisms (SNPs) in pre-miRNAs can alter miRNA processing and maturation, while SNPs in mature miRNA regions can modify the binding affinity between miRNAs and their mRNA targets. These variations are associated with different aspects of colorectal cancer, including risk, prognosis, protection, and response to treatment, highlighting their role as potential biomarkers in understanding and managing the disease. Created with BioRender.com (accessed on 25 November 2024). The colon and cell proliferation in this figure were modified from Servier Medical Art [58], licensed under a Creative Commons Attribution 3.0 Generic License [59].

Figure 2 summarizes changes in miRNA expression induced by SNPs and their influence on colorectal cancer.

### 4.1. miR-608 rs4919510

Two studies [39,47] found significant associations between the SNP rs4919510 in mature miR-608 and the prognosis of CRC patients. The study by Xing et al. demonstrated that rs4919510 in pre-miR-608 is associated with changes in recurrence-free survival in patients undergoing chemotherapy [57]. In contrast, another study indicated that the rs4919510 G allele and the rs4919510 GG genotype are associated with decreased susceptibility to CRC, observed in codominant, superdominant, and recessive (GG vs. CC) models, suggesting a protective effect among stage 0-II cases in the Chinese population [60]. This evidence corroborates what was observed in the study by Pardini et al., which revealed that individuals carrying the SNP rs4919510 G in miR-608 had a reduced risk of recurrence compared with those carrying the wild-type genotype [61]; however, further studies are needed to investigate the impact of the SNP rs4919510 on different regions of miR-608 and its relationship with CRC prognosis.

### 4.2. miR-618 rs2682818/rs1051690

Two studies have associated the rs2682818 SNP in mature miR-618 with reduced CRC risk, especially in patients with the rs2682818 A>C genotype [42,44]. In contrast, Shao et al. demonstrated that the rs2682818 SNP is associated with CRC risk, observing that individuals with the rs2682818 GG/TG genotypes had worse overall survival and reduced expression of mature miR-618 compared with those with the homozygous rs2682818 TT genotype [62]. In the study by Landi et al., the SNP rs1051690 in the miR-618 binding site was associated with an increased risk of CRC, especially in patients with the rs1051690 G>A genotype [25]. To date, no other studies investigating the SNP rs1051690 in the context of miR-618 have been identified; therefore, additional studies are needed to evaluate the functional effects of this SNP on miR-618 and its possible relationship with CRC susceptibility. Future investigations may contribute to improving risk stratification and support the development of more effective strategies for disease screening and prevention.

### 4.3. miR-196a2 rs11614913

Three studies found no significant association between the SNP rs11614913 T>C in mature miR-196a2 and CRC risk in two distinct populations [33,48,49]. In contrast, Zhan et al. reported that Chinese individuals with the rs11614913 CC genotype or carrying at least one C allele of the SNP rs11614913 had higher levels of mature miR-196a and were associated with a higher risk of CRC, although no association with cancer progression was observed [40]. Furthermore, another study indicated that the C>T genotype of the SNP rs11614913, located in the hsa-mir-196a2 pre-miRNA, was associated with an increased risk of CRC compared with the rs11614913 CC genotype [63]. On the other hand, a meta-analysis suggested that the TT genotype of the SNP rs11614913 might be associated with reduced cancer risk, especially for CRC and lung cancer, in Korean and Indian populations [64]. These contradictory findings highlight the need for further investigation to understand better the impact of the SNP rs11614913 on miR-196a2 and its relationship with CRC risk.

### 4.4. miR-149 rs2292832

Chayeb et al. reported that the SNP rs2292832 C>T in miR-149 and rs2910164 G>C in miR-146a are associated with CRC risk [33]. A meta-analysis highlighted that individuals with the rs2292832 TT genotype are at increased risk of developing CRC compared with those who carry at least one rs2292832 C allele [65]. Furthermore, another study revealed that the rs2292832 TT genotype is associated with an increased risk of breast cancer. Located in pre-mir-149, rs2292832 may alter the processing of miR-149, affecting the abundance of its mature form. These alterations may play a crucial role in tumor progression and metastasis [66].

### 4.5. miR-146a rs2910164

The SNP rs2910164 G>C in the miR-146a region has been associated with CRC risk and progression in two studies [33,35]. Iguchi et al. reported a significant association between the rs2910164 CC/CG genotypes in pre-miR-146a and liver metastasis [67]. Another study analyzing four SNPs in the pre-miRNA region demonstrated that the rs2910164 G>C genotype was associated with an increased risk of CRC, while the rs2910164 CC genotype was associated with a decreased risk [63]. Furthermore, a meta-analysis revealed that the C allele of rs2910164 is associated with a decreased risk of cancer in the Chinese population [64]. Another study in the same population indicated that the rs2910164 GG genotype or rs2910164 G allele was associated with an increased risk of CRC, especially in men, when compared with the rs2910164 CC genotype or the rs2910164 C allele [56]. These results suggest that miR-146a may play distinct roles depending on the individual’s genotype, highlighting the need for further research to understand these variations fully.

### 4.6. miR-27a rs895819

The rs895819 SNP is located in the terminal loop of pre-miR-27a and influences its secondary structure, resulting in abnormal expression of hsa-miR-27a [68]. In a study conducted by Cao et al., it was shown that the GG genotype of rs895819 in miR-27a is significantly associated with an increased risk of CRC and metastasis, compared with the rs895819 AA genotype in the Chinese population [34]. Similar results were observed in an independent study in the same population, which suggested that the GG genotype of rs895819 was associated with an increased risk of CRC, while the rs895819 G allele was associated with an increased likelihood of disease progression [69]. Another study conducted by Wang et al. corroborated these findings, showing that the GG genotype and G allele of rs895819 were significantly associated with an increased risk of CRC in the same population [20]. On the other hand, a study conducted in a European population found no association between the rs895819 T>C SNP in miR-27a and the risk of CRC [70]. Another study conducted in the same population demonstrated that individuals with the rs895819 A (AA/GA) allele had a reduced risk of CRC [68]. These results reinforce the need for additional studies that include populations of different ethnic origins to understand better the impact of this SNP on the development of CRC.

### 4.7. miR-211 rs187960998

A single study identified the rs187960998 SNP in mature miR-211 as a tumor suppressor, demonstrating its ability to inhibit CC cell proliferation and invasion through upregulation of CHD5 [43]. Other studies have focused on miR-211 expression and its role in CRC without establishing a clear association with the presence of SNPs [71,72]; therefore, further investigations are needed to understand better the impact of this SNP in miR-211 in the context of cancer.

### 4.8. miR-4707 rs2273626 and miR-4274 rs202195689

The current literature on the SNP rs2273626 in miR-4707 and rs202195689 in miR-4274 in the context of cancer is limited. Huang et al. associated these SNPs, located in miRNA seed regions, with a reduced risk of grade 2 leukopenia, as well as improved overall survival and disease-free survival [41]. Ghanbari et al. demonstrated that the SNP rs2273626 in miR-4707 influences the interaction with the glaucoma-related target gene Caspase Recruitment Domain Family Member 10 (CARD10), resulting in elevated levels of this gene [73].

### 4.9. miR-525-5p rs8176318

The study by Mullany et al. highlighted that the rs8176318 AA SNP, located in the 3′-UTR of the BRCA1 gene and predicted to bind to miR-525-5p, is associated with an increased risk of CC [32]. On the other hand, Cao et al., who suggested that the G>T genotypes or individuals carrying the T allele of the rs8176318 SNP could be associated with an increased risk of breast cancer compared with the GG genotype [74]. These evidences highlight the need further investigations to elucidate the functional role of miR-525-5p and the rs8176318 SNP in the 3′-UTR region of BRCA1, as well as their influence on different genotypes and types of cancer.

### 4.10. miR-200c rs12904

A study in the Iranian population revealed that the rs12904 AA genotype is associated with CRC pathogenesis and metastasis, while the rs12904 A>G genotype is related to reduced disease progression. Furthermore, the SNP rs12904 in the EFNA1 gene influences the expression of this gene, mediated by the miR-200c and miR-429a [37]. In contrast, Simonian et al. found no association between the SNP rs12904 and the risk of CRC in the same population [50]. On the other hand, a study carried out in the Chinese population identified that the SNP rs12904 located in the miR-200c binding site of the EFNA1 gene is associated with an increased risk of developing gastric cancer, with the rs12904 AA genotype correlating with a higher expression of EFNA1 in gastric tissues, although it has no effect on the expression of the miRNA [75].

### 4.11. let-7 KRAS-LCS6/rs868

Studies by Smits et al. and Xicola et al. investigated the KRAS-LCS6 SNP of the KRAS gene and rs868 of the TGFBR1 gene in the miRNA let-7 binding site. The results showed that the KRAS-LCS6 SNP is associated with the prognosis of CRC, while the rs868 SNP is related to the risk of the disease [31,38]. Furthermore, another study indicated that mCRC patients carrying the LCS6 SNP had a better response to therapy with anti-EGFR monoclonal antibodies (moAbs) [76]. On the other hand, Christensen et al. demonstrated that patients with head and neck squamous cell carcinoma carrying the SNP KRAS-LCS6 had significantly reduced survival time, suggesting that the presence of this SNP may alter the phenotype or therapeutic response in this condition [77]. The rs868 SNP, located in the 3′-UTR of the TGFBR1 gene, has been shown to have a regulatory effect on miRNA/mRNA interaction by influencing negative RNA correlation in patients with hepatitis C-associated end-stage liver disease. Furthermore, these SNPs affect the expression of miRNAs let-7 and miR-98 after liver transplantation, with a particularly significant effect in carriers of the rs868 A>G genotype [78]. Despite the associations identified between the KRAS-LCS6 and rs868 SNPs in different aspects of CRC and other diseases, several open questions remain. Additional studies are needed to understand better the mechanisms by which these SNPs influence risk, prognosis, and response to treatment.

Despite advances in the understanding of miRNAs, important gaps remain in the scientific literature. Genomic instability, a central hallmark of cancer, can be influenced by miRNAs, but the mechanisms that link these small non-coding RNAs to cell cycle regulation, DNA damage response, and mitotic separation are still not fully understood. This emerging area is critical to understanding how miRNAs may contribute to cancer initiation and progression but requires further experimental investigation to unravel these complex pathways [79,80]

Another significant hurdle lies in the development of miRNA-based therapeutics. The biogenesis and delivery of miRNAs to target cells, particularly using extracellular vesicles such as exosomes, shows promise, but limitations related to efficiency and specificity remain. The high redundancy of miRNAs and their wide range of targets increase the risk of off-target effects, posing additional challenges for their clinical application [80,81,82].

Despite this, miRNAs continue to show enormous potential as clinical tools; however, bridging the gaps in understanding their biological mechanisms, standardizing methods, and developing safe therapeutic strategies are essential to maximize their impact on cancer treatment [79,80].

### 4.12. Limitations and Future Perspectives

This review presents some limitations that should be considered when interpreting the results. In addition to the scarcity of studies focused on SNPs in specific miRNA regions for CRC, we observed significant heterogeneity among the included studies. Most participants were predominantly Caucasian or Asian, which may introduce genetic and geographic variations that influence the findings. Despite these limitations, a notable advantage of this systematic review is the ability to evaluate, based on the existing literature and studies of high methodological quality, the hypothetical associations of SNPs in miRNA regulatory regions and their potential as biomarkers for CRC.

## 5. Conclusions

In recent years, the study of SNPs in miRNA regions has aroused great interest in the search for new promising, less invasive, sensitive, and specific biomarkers for the early detection of CRC. This systematic review provides valuable insights into studies related to the diagnosis, prognosis, and treatment of CRC, focusing on mirSNPs and contributing to a better understanding of the pathways involved in the development of the disease. Our findings highlight the potential of SNPs in miRNAs as important tools in the early identification and management of CRC.

## Figures and Tables

**Table 2:** Association of SNPs in miRNA regions with colorectal cancer risk.

Study	Population	Number of Cases/Controls	SNP	Gene and Target miRNA	Results
[25]	Czech Republic	697/624	rs17281995, rs1051690	CD86, INSR	Increased CRC risk due to alteration in miRNA binding site
[26]	China	386/394	rs141178472	PIK3CA (miR-520a)	CC genotype associated with increased CRC risk
[27]	China	1033/1147	rs3814058	PXR (hsa-miR-129-5p)	Gene expression alteration, increasing CRC risk
[28]	China	1147/1203	rs6504593	IGF2BP1 (miR-21)	T allele affects gene expression and increases CRC risk
[29]	China	2347/3390	rs1062044	LAMC1 (miR-423-5p)	A allele increases CRC risk
[30]	Korea	325/977	rs7930	TOMM20 (miR-4273-5p)	A allele is protective, while the G allele increases CRC risk due to interaction with miR-4273-5p
[31]	Spain	723/629	rs868	TGFBR1 (miR-let-7b-5p)	Increased CRC risk due to decreased TGFBR1 expression
[32]	USA	1115/1173	rs8176318, rs8905	BRCA1 (miR-525-5p), PRKAR1A (miR-214-3p)	Increased risk of colon cancer
[33]	Tunisia	152/161	rs2292832, rs2910164	hsa-miR-149, hsa-miR-146a	Significant association with CRC risk (rs2292832, rs2910164)
[34]	China	254/238	rs895819	miR-27a	Increased CRC risk with AG/GG genotypes

**Table 3:** Association of SNPs in miRNA regions with colorectal cancer prognosis.

Study	Population	Number of Cases/Controls	SNP	Gene and Target miRNA	Results
[35]	China	276/373	rs2910164	miR-146a	Associated with CRC susceptibility and differentiation
[36]	Korea	831 CRC cases	rs3757417, rs12373	TPST1, PAUF (miR-571)	Potential prognostic marker in surgical resection. Increased miR-571 affinity, impacting survival
[37]	Iran	146/132	rs12904	EFNA1, miR-200C, miR-429	AA genotype linked to pathogenesis and metastasis, A>G genotype associated with lower progression
[38]	Netherlands	Large sample (Biobank)	LCS6	KRAS (let-7)	Association of LCS6 T>G with improved early-stage CRC survival
[39]	Caucasians and African Americans	245/446	rs4919510	miR-608	GG genotype associated with higher mortality risk in Caucasians, lower in African Americans
[40]	China	252/543	rs11614913	miR-196a2	CC genotype associated with higher prevalence and elevated mature miR-196a2 levels, linked to susceptibility
[41]	China	365 rectal cancer patients	rs2273626, rs202195689	miR-4707-3p, miR-4274	rs2273626 associated with lower leukopenia risk, rs202195689 with overall and disease-free survival

**Table 4:** Association of SNPs in miRNA regions with risk reduction, protection, and treatment of colorectal cancer.

Study	Population	Number of Cases/Controls	SNP	Gene and Target miRNA	Results
[42]	Korea	895/901	rs2279398	DOK3 (miR-370)	Associated with risk reduction through regulation of the DOK3 gene
[43]	China	878/884	rs2682818	miR-618	AA and AC/AA genotypes associated with reduced CRC risk
[44]	China	685/618	rs187960998	CHD5 (miR-211)	Associated with reduced CRC risk and favorable clinical characteristics
[45]	Bulgaria	104 mCC cases	rs2682818	miR-618	Heterozygous AC genotype associated with significantly lower mCC risk compared with CC and AA genotypes
[46]	China	685/618	rs35301225	E2F1 (miR-34a)	rs35301225 may act as a tumor suppressor by negatively regulating E2F1; however, C>A genotype is associated with higher E2F1 expression and worse survival
[47]	Multicenter study	155 LARC patients	rs4919510	miR-608	CC genotype associated with worse prognosis in LARC patients treated with perioperative CAPOX, chemoradiotherapy, and surgery

**Table 5:** Studies of SNPs in miRNA regions with no significant association with colorectal cancer.

Study	Population	Number of Cases/Controls	SNP	Gene and miRNA Target	Results
[48]	China	126/407	rs11614913	miR-196a2	No significant association
[49]	Iran	52/120	rs11614913	miR-196a2	No significant association
[50]	Iran	152/160	rs12904	EFNA1 (miR-200c)	No significant association

## Data Availability

Data that support the findings of this study are available from the corresponding author upon reasonable request.

## Supplementary Materials

The following supporting information can be downloaded at: https://www.mdpi.com/article/10.3390/ijms252312975/s1.
