# Supplementary material for: mirSNPs as Potential Colorectal Cancer Biomarkers: A Systematic Review
Source: Int J Mol Sci. 2024 Dec 3;25(23):12975. doi: 10.3390/ijms252312975 (PMC11641632; doi:10.3390/ijms252312975)
Supplement: Supplementary file 1 [file ijms-25-12975-s001.zip › Table S2.MMAT.pdf]

**Table S2.** Assessment of the quality of studies evaluated by the Mixed Methods Assessment Tool

| Category of study designs | Methodological quality criteria | Responses | | | |
|---|---|---|---|---|---|
| | | Yes | No | Can't tell | Study |
| Screening questions (for all types) | S1. Are there clear research questions? | ✓ | | | |
| | S2. Do the collected data allow addressing the research questions? | ✓ | | | |
| | Further appraisal may not be feasible or appropriate when the answer is 'No' or 'Can't tell' to one or both screening questions. | | | | |
| 1. Qualitative | 1.1. Is the qualitative approach appropriate to answer the research question? | | | | |
| | 1.2. Are the qualitative data collection methods adequate to address the research question? | | | | |
| | 1.3. Are the findings adequately derived from the data? | | | | |
| | 1.4. Is the interpretation of results sufficiently substantiated by data? | | | | |
| | 1.5. Is there coherence between qualitative data sources, collection, analysis and interpretation? | | | | |
| 2. Quantitative randomized controlled trials | 2.1. Is randomization appropriately performed? | ✓ | | | |
| | 2.2. Are the groups comparable at baseline? | ✓ | | | |
| | 2.3. Are there complete outcome data? | ✓ | | | [47] |
| | 2.4. Are outcome assessors blinded to the intervention provided? | | | | |
| | 2.5 Did the participants adhere to the assigned intervention? | ✓ | | | |
| 3. Quantitative non-randomized | 3.1. Are the participants representative of the target population? | ✓ | | | |
| | 3.2. Are measurements appropriate regarding both the outcome and intervention (or exposure)? | ✓ | | | |
| | 3.3. Are there complete outcome data? | ✓ | | | [25,26,27,28,29,30,31,32,33,34,35,36,37,38,39,40,41,42,43,44,45,46,48,49,50] |
| | 3.4. Are the confounders accounted for in the design and analysis? | ✓ | | | |
| | 3.5. During the study period, is the intervention administered (or exposure occurred) as intended? | ✓ | | | |
| 4. Quantitative descriptive | 4.1. Is the sampling strategy relevant to address the research question? | | | | |
| | 4.2. Is the sample representative of the target population? | | | | |
| | 4.3. Are the measurements appropriate? | | | | |
| | 4.4. Is the risk of nonresponse bias low? | | | | |
| | 4.5. Is the statistical analysis appropriate to answer the research question? | | | | |

| | |
|---|---|
| 5. Mixed methods | 5.1. Is there an adequate rationale for using a mixed methods design to address the research question?<br>5.2. Are the different components of the study effectively integrated to answer the research question?<br>5.3. Are the outputs of the integration of qualitative and quantitative components adequately interpreted?<br>5.4. Are divergences and inconsistencies between quantitative and qualitative results adequately addressed?<br>5.5. Do the different components of the study adhere to the quality criteria of each tradition of the methods involved? |
